# Antiviral activity of itraconazole against type I feline coronavirus infection

**DOI:** 10.1186/s13567-019-0625-3

**Published:** 2019-01-18

**Authors:** Tomomi Takano, Misuzu Akiyama, Tomoyoshi Doki, Tsutomu Hohdatsu

**Affiliations:** School of Veterinary Medicine, Kitasato University, Towada, Aomori Japan

## Abstract

Feline coronaviruses (FCoVs) are the causative agents of severe systemic disease (feline infectious peritonitis: FIP) in domestic and wild cats. FCoVs have been classified into serotypes I and II. Type I FCoV is the dominant serotype (approximately 70–90%) worldwide. Therefore, it is necessary to provide antiviral agents for type I FCoV infection. In this study, we demonstrated that itraconazole (ICZ), practically used for fungal infections in cats, inhibits the type I FCoV infection. ICZ also exhibited antiviral effect in cells after viral infection, suggesting that ICZ could potentially be used as a therapeutic.

## Introduction, methods and results

Feline coronavirus (FCoV; family *Coronaviridae*, genus *Alphacoronavirus*) is an enveloped, single-stranded, positive-sense RNA virus. FCoV exists as two different biotypes: feline enteric coronavirus (FECV) and feline infectious peritonitis virus (FIPV) [1]. The former causes mild enteritis (usually subclinical infection), and the latter causes the highly lethal systemic disease FIP. Although antiviral drugs and vaccines against FIP have been investigated, no method has been established for practical use [2]. Furthermore, FCoV also exists as two serotypes: type I FCoV (type I FECV and type I FIPV) and type II FCoV (type II FECV and type II FIPV) [3]. Serological and genetic surveys showed that type I FCoV is dominant worldwide [4–6].

We previously reported that type I FCoV is closely associated with cholesterol throughout the viral life cycle [7]. We also demonstrated that U18666A, the cholesterol transport inhibitor, strongly inhibits type I FCoV infection [8]. Based on these findings, U18666A may be applied as a therapeutic drug for FIP. However, to our knowledge, U18666A is not approved for veterinary practical use. To use U18666A to treat FIP, pharmacokinetic, pharmacodynamic, and safety studies must be performed for cats. As with U18666A, several candidate antiviral drugs targeting type I FCoV have been identified [9, 10]. However, none of drugs scientifically demonstrated to exhibit a therapeutic effect on FIP are practically used. To solve these problems, it is desirable to identify a potent antiviral agent for FCoV infection among drugs generally used for cats.

Itraconazole (ICZ) is classified as an azole antifungal [11]. It has low toxicity and can be used to treat fungal infections in immunocompromised patients [12]. It is commonly used by veterinarians to treat fungal infections in dogs and cats. Recently, ICZ was suggested to be effective for enteroviral infection (poliovirus, rhinovirus, and coxsackievirus) [13]. We previously investigated the antiviral effects of cholesterol transport inhibitors, including U18666A, and confirmed that ICZ inhibits type I FCoV infection [8]. However, the influence of ICZ on FCoV infection has not been investigated in detail. In this study, we examined the antiviral effects of ICZ on FCoV.

**Figure 1:**
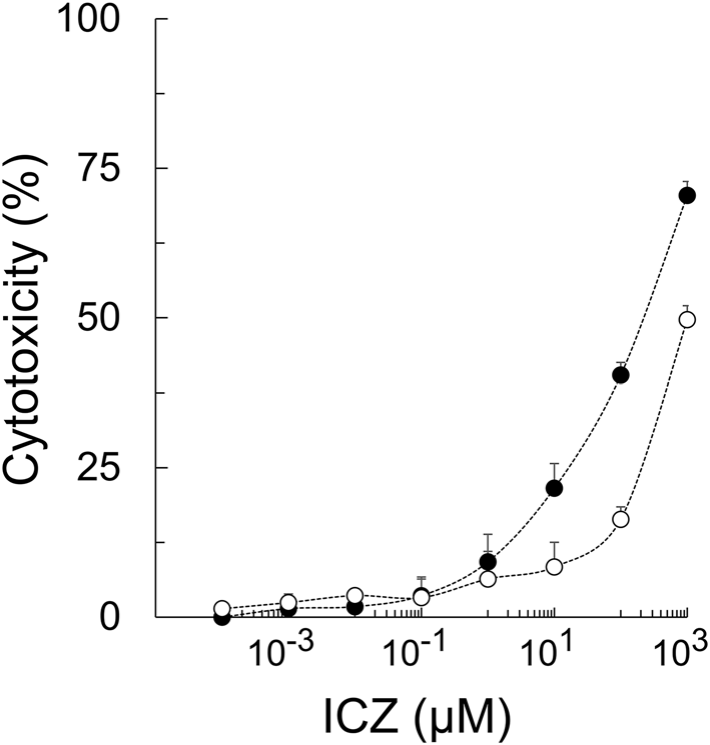
**Cytotoxic effects of ICZ in fcwf-4 cells.** fcwf-4 cell viability was measured by the WST-8 assay as a described before [7]. The black circles indicate treatment with ICZ and the white circles indicate treatment with the solvent (solvent control). The solvent concentration was the same as that in ICZ solution at each serial dilution. Data represent three independent experiments.

Felis catus whole fetus (fcwf)-4 cells sensitive to type I FIPV, type II FIPV, and type II FECV were used. ICZ (ITORIZOLE^®^) was purchased from Janssen Pharmaceutical K.K. (Tokyo, Japan). For the solvent of ICZ, 40% (w/v) hydroxypropyl-beta-cyclodextrin (FUJIFILM Wako Pure Chemical, Japan) containing 2.5% (v/v) propylene glycol and 0.376% (v/v) hydrochloric acid was used. ICZ was adjusted to 10 mM with the solvent, aliquoted, and stored at −30 °C until use. Maintenance medium (MEM) was used to dilute ICZ. To evaluate the cytotoxic effects of ICZ and solvent in fcwf-4 cells, cell viability was measured by the WST-8 assay as a described before [7]. The percent cytotoxicity was calculated using the following formula: Cytotoxicity (%) = 100 − [(OD of ICZ (or solvent)-treated cells/OD of ICZ (or solvent)-untreated cells)] × 100. The final pH of all the diluents of ICZ and solvent was 7.5–7.6. The 50% cytotoxic concentration (CC_50_) and 10% cytotoxic concentration (CC_10_) values of ICZ were 208.0 ± 22.9 (mean ± SE) μM and 1.1 ± 0.4 (mean ± SE) μM, respectively (Figure 1).

**Figure 2:**
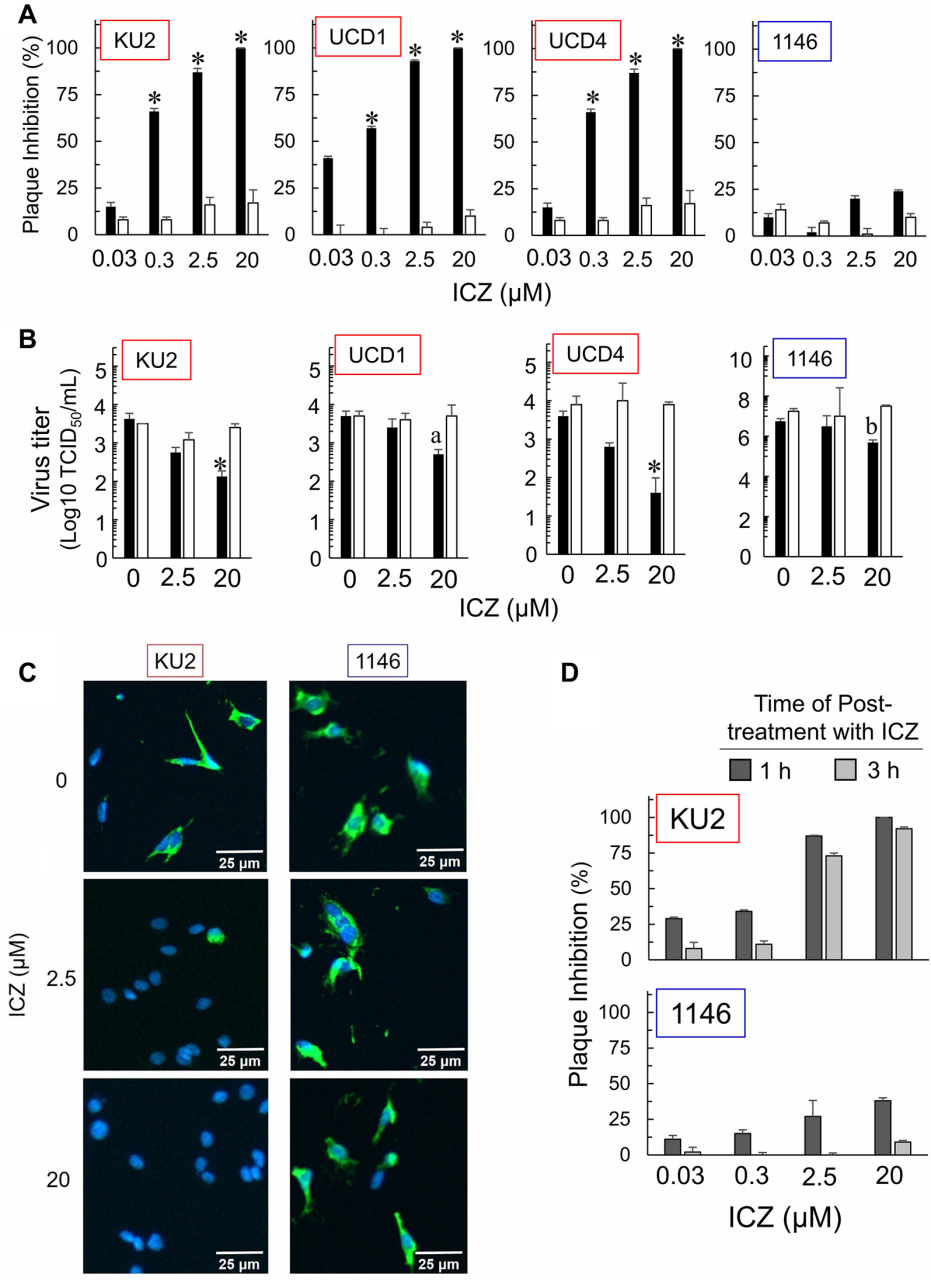
**Antiviral effects of ICZ on type I FIPV, and type II FIPV.** **A** Plaque inhibition assay of FCoV in fcwf-4 cells treated with ICZ. The black bars indicate treatment with ICZ and the white bars indicate treatment with the solvent (solvent control). The results are shown as the mean ± SE. Data represent four independent experiments. *p < 0.05 v.s. solvent control. **B** Inhibition of FCoV infection by ICZ in fcwf-4 cells. The black bars indicate treatment with ICZ and the white bars indicate treatment with the solvent (solvent control). The results are shown as the mean ± SE. Data represent four independent experiments. *p < 0.05 v.s. solvent control. a: *p* = 0.054 v.s. solvent control. b: *p* = 0.082 v.s. solvent control. c: *p* = 0.052 v.s. solvent control. **C** Effects of ICZ on FCoV nucleocapsid (N) expression. FCoV N protein was evaluated by IFA. Data represent three independent experiments. **D** Effects of post-treatment on the antiviral activity of ICZ. KU2: FIPV-I KU2 strain, UCD1: FIPV-I UCD1 strain, UCD4: FIPV-I UCD4 strain, 1146: FIPV-II WSU 79-1146 strain, ICZ: itraconazole.

The influence of ICZ on infection of 3 strains of type I FCoV (FIPV-I KU2, FIPV-I UCD1, and FIPV-I UCD4), 1 strain of type II FCoV (FIPV-II WSU79-1146) in fcwf-4 cells was investigated. Confluent fcwf-4 cell monolayers were cultured in medium containing ICZ at the indicated concentrations in 24-well multi-plates at 37 °C for 24 h. Cells were washed and the virus (MOI 0.01) was adsorbed onto the cells at 37 °C for 1 h. After washing, cells were cultured in carboxymethyl cellulose (CMC)-MEM or MEM without CMC. To perform plaque inhibition assay using a 24-well plastic plate, it is necessary to clearly prepare a plaque in a small area of monolayer cells, for which CMC-MEM is more appropriate than agar-MEM for overlay medium. For the plaque inhibition assay, cells cultured in CMC-MEM were incubated at 37 °C for 48 h, fixed and stained with 1% crystal violet solution containing 10% buffered formalin, and the resulting plaques were then counted. The percentage of plaque inhibition was calculated using the following formula: Percentage of plaque inhibition (%) = 100 − [(plaque number by compound-treated cells)/(plaque number by compound-untreated cells)] × 100. For cells cultured in MEM, the culture supernatants were collected at 48 h post-infection, and the virus titer was determined by titration assay. Pre-treatment with ICZ reduced plaque formation by type I FCoVs in a dose-dependent manner (Figure 2A). Plaque formation by type I FCoVs were inhibited by 0.03–20 μM ICZ. In contrast, the percentage of plaque inhibition for type II FCoVs was slightly affected by pre-treatment with ICZ. IC50 and SI (CC50/IC50) of ICZ for each virus based on the results of plaque inhibition assay and cytotoxicity assay are shown in Table 1. According to the titration assay, the production of type I FCoVs were dose-dependently decreased by ICZ (Figure 2B). The production of type II FCoVs were slightly decreased only when 20 μM ICZ was added. We next investigated the expression of viral proteins in order to further evaluate the effects of ICZ on FCoV infection. Fcwf-4 cells were grown on an 8-well Lab-Tek Chamber Slide (Thermo Fisher Scientific, USA). Confluent fcwf-4 cell monolayers were cultured in medium containing 20 μM ICZ at 37 °C for 24 h. Cells were washed and the virus (MOI 0.01) was adsorbed into the cells at 37 °C for 1 h. After washing, cells were cultured in MEM. The cell monolayers were incubated at 37 °C. After 72 h, nucleocapsid protein levels were determined by immunofluorescence assay (IFA), as described previously [14]. To recognize FIPV N protein, mAb YN-2 (mouse IgG2b) prepared by our laboratory was used [15]. Nuclei were stained with 4′,6-diamidino-2-phenylindole (DAPI; Dojindo laboratories, JAPAN). The N protein levels of FIPV-I KU2 were specifically decreased in fcwf-4 cells pre-treated with ICZ. In contrast to FIPV-I KU2, pre-treatment with ICZ did not affect the N protein levels of FIPV-II 79-1146 in fcwf-4 cells (Figure 2C).

**Table 1:** **The CC50, IC50, and SI of itraconazole**

Virus/cell	CC50 (μM)	IC50 (μM)	SI (CC50/IC50)
FIPV-I KU2	–	0.219	950.6
FIPV-I UCD1	–	0.597	348.7
FIPV-I UCD4	–	0.146	1425.9
FIPV-II 79-1146	–	> 160.0	< 1.3
fcwf-4 cell	208.0	–	–

Furthermore, we examined the effects of ICZ on plaque formation after infection of fcwf-4 cells with FIPV-I KU2. The virus at MOI of 0.01 was added to the culture and adsorbed by fcwf-4 cells at 37 °C for 1 h. After washing, cells were cultured in MEM at 37 °C for 1 or 3 h. After exchanging MEM for CMC-MEM containing ICZ, cells were cultured at 37 °C for 48 h. The percentage of plaque inhibition was measured as described above. Post-treatment with ICZ inhibited FIPV-I KU2 plaque formation to a degree comparable with pre-treatment (Figure 2D). In contrast, the percentage of plaque inhibition by FIPV-II 79-1146 was slightly affected by post-treatment with ICZ.

**Figure 3:**
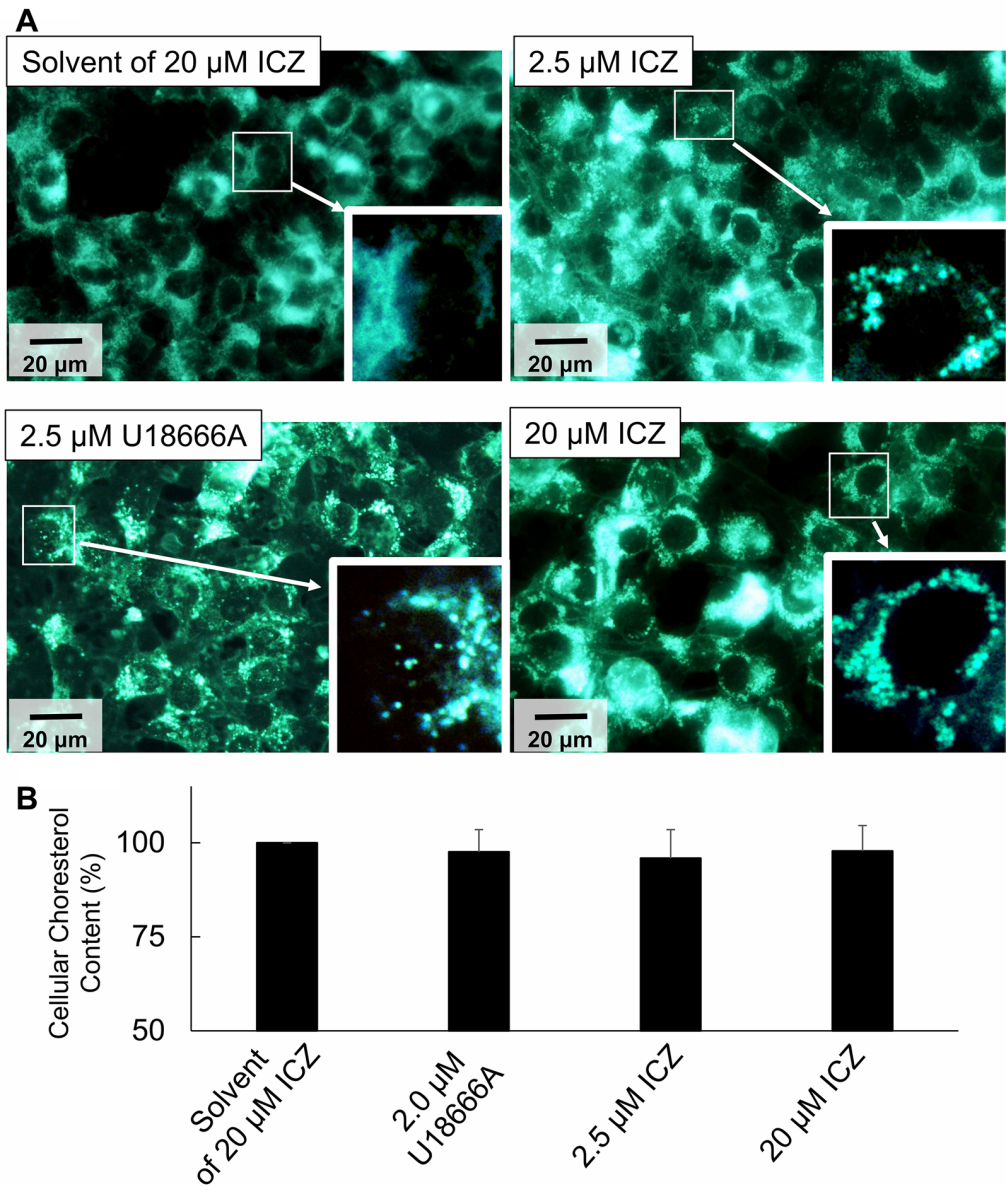
**Effect of ICZ on cholesterol accumulation and cellular cholesterol level in fcwf-4 cells.** **A** ICZ induced accumulation of intracellular cholesterol. The cellular cholesterol content in cells was evaluated by filipin-cholesterol staining. The area within the white square is magnified in the cell indicated by white arrow. **B** Quantification of cellular cholesterol in fcwf-4 cells. Data represent three independent experiments.

Type I FCoVs infection are strongly inhibited by the cholesterol transport inhibitor U18666A [8]. ICZ has been reported to inhibit intracellular cholesterol transport, similar with U18666A [16]. Therefore, we assessed whether ICZ inhibits intracellular cholesterol transport in cat cells. The cellular cholesterol content in fcwf-4 cells was evaluated using the Cholesterol Cell-Based Detection Assay Kit (Cayman chemical, USA) according to the manufacturer’s instructions. Briefly, fcwf-4 cells were grown on an 8-well Lab-Tek Chamber Slide (Thermo Fisher Scientific, USA). Semi-confluent fcwf-4 cell monolayers were cultured in medium containing 20 μM ICZ or 2 μM U18666A (FUJIFILM Wako Pure Chemical, Japan) at 37 °C for 24 h. After fixing and staining, filipin III-stained cells were analyzed using a Leica DM4B microscope and LAS X integrated imaging system (Leica Microsystems, Germany). ICZ induced accumulation of cellular cholesterol, similar with U18666A (Figure 3A). To determine whether ICZ affected the cellular cholesterol content, the level of cholesterol in fcwf-4 cells was measured. The amount of intracellular cholesterol was determined using the Amplex Red Cholesterol Assay Kit (Molecular Probes, USA) following the manufacturer’s instructions. Neither 2.5 μM nor 20 μM ICZ decreased cellular cholesterol levels as with 2.0 μM U18666A (Figure 3B).

## Discussion

Of the 2 serotypes, ICZ inhibited type I FCoV infection. As approximately 70–90% of cats with FIP are infected with type I FCoV [4–6], it would be reasonable to test the use ICZ as an anti-FIPV agent. On the other hand, ICZ did not affect type II FCoV, suggesting that the antiviral effects of ICZ differ depending on the serotype of FCoV. The incidence of type II FIPV-induced FIP in Japan and Taiwan is higher than that in Europe [6, 17, 18], and the combination of ICZ with other drugs is necessary in these countries.

A difference was noted between the results of plaque inhibition and virus titration assays in virus-infected cells treated with 2.5 μM ICZ: a significant inhibitory effect on type I FIPV infection was observed in the former but no significant inhibitory effect was noted in the latter. Since plaque inhibition assay measures the infectivity titer of parental virus, whereas virus titration assay measures that of progeny virus, these findings suggest that 2.5 μM ICZ strongly inhibited parental virus infection but the concentration was insufficient to inhibit progeny virus production. However, the titer of produced progeny virus and results of plaque inhibition assay strongly suggest that progeny virus infection is inhibited in cells treated with 2.5 μM ICZ.

Boothe et al. [19] previously reported the pharmacokinetic variables of ICZ in cats. According to their report, the peak blood concentration (Cmax) was 1.1 ± 3.6 μg/mL (1.6 ± 5.1 μM) in cats treated with oral ICZ at 10 mg/kg once, and it reached 3.4 ± 1.3 μg/mL (4.8 ± 1.8 μM) in cats treated with oral ICZ twice within a 12-h interval. In the present study, plaque formation and viral antigen expression were inhibited in fcwf-4 cells treated with 2.5 μM ICZ, and similar effects were noted in cells treated with ICZ after infection with FIPV-I KU2, suggesting that administration of ICZ at 10 mg/kg twice daily to cats diagnosed with FIP may decrease the viral load. For the treatment of fungal infection in cats, ICZ is administered at a high dose (up to 26.7 mg/kg) in the early phase [20]. Administration of ICZ at a high dose in the early phase may also exert antiviral effects against FIP. Blood alanine aminotransferase has been reported to increase when high-dose ICZ is continued, but this symptom improved after discontinuing drug administration [20]. We recommend that veterinarians use ICZ for treatment of FIP following the fungal infection treatment protocol in cats.

We previously reported that U18666A inhibits cholesterol transport and type I FIPV infection by acting on a cholesterol transporter, Niemann-Pick C1 protein (NPC1) [8]. In this study, inhibition of intracellular cholesterol transport by ICZ was confirmed. Accumulation of cholesterol in the cytoplasm induced by ICZ has been reported by several studies [14, 21] other than the present study, but the mechanism has not been clarified. It has recently been reported that ICZ induces cholesterol accumulation in lysosome by acting on NPC1. Based on this report, ICZ may inhibit type I FCoV replication via the same mechanism as U18666A. In addition, in viruses other than type I FCoV, ICZ inhibits infection by acting on proteins other than NPC1. For example, it has been reported that ICZ acts on oxysterol-binding protein (OSBP) and inhibits formation of the viral replication organelle in enteroviruses [14]. The site of replication of Coronavirus is presumed to be associated with endoplasmic reticulum (ER)-derived structures often referred to as double membrane vesicles (DMVs) [22], but it is unclear whether OSBP is involved in the formation of the viral replication organelle in coronavirus. The relationship between the infection of type I FIPV and OSBP needs to be investigated.

In this study, we confirmed that ICZ inhibits infection by type I FIPV, the dominant strain in the field, suggesting that ICZ can be applied as a therapeutic drug for FIP. FIP is a “multi-causal disease” involving various risk factors (virulence of FCoV, the status of immunity in host, and the route of virus infection etc…). Taking this fact into consideration, we are planning to perform a clinical trial of ICZ using cats diagnosed with FIP and investigate combination with other therapeutic drugs for FIP at the same time.

## Abbreviations

FCoV: feline coronavirus
FIP: feline infectious peritonitis
FECV: feline enteric coronavirus
ICZ: itraconazole
fcwf-4: Felis catus whole fetus-4
MEM: maintenance medium
CMC: carboxymethyl cellulose
CC_50_: 50% cytotoxic concentration
MOI: multiplicity of infection
TCID_50_: median tissue culture infective dose
IFA: immunofluorescence assay
NPC1: Niemann-Pick C1
